# Development of a Tetrameric Streptavidin Mutein with Reversible Biotin Binding Capability: Engineering a Mobile Loop as an Exit Door for Biotin

**DOI:** 10.1371/journal.pone.0035203

**Published:** 2012-04-19

**Authors:** Valerie J. O'Sullivan, Isabelle Barrette-Ng, Eric Hommema, Greg T. Hermanson, Mark Schofield, Sau-Ching Wu, Claudia Honetschlaeger, Kenneth K.-S. Ng, Sui-Lam Wong

**Affiliations:** 1 Thermo Fisher Scientific, Inc., Pierce Protein Research, Rockford, Illinois, United States of America; 2 Department of Biological Sciences, University of Calgary, Calgary, Alberta, Canada; University of Houston, United States of America

## Abstract

A novel form of tetrameric streptavidin has been engineered to have reversible biotin binding capability. In wild-type streptavidin, loop_3–4_ functions as a lid for the entry and exit of biotin. When biotin is bound, interactions between biotin and key residues in loop_3–4_ keep this lid in the closed state. In the engineered mutein, a second biotin exit door is created by changing the amino acid sequence of loop_7–8_. This door is mobile even in the presence of the bound biotin and can facilitate the release of biotin from the mutein. Since loop_7–8_ is involved in subunit interactions, alteration of this loop in the engineered mutein results in an 11° rotation between the two dimers in reference to wild-type streptavidin. The tetrameric state of the engineered mutein is stabilized by a H127C mutation, which leads to the formation of inter-subunit disulfide bonds. The biotin binding kinetic parameters (k_off_ of 4.28×10^−4^ s^−1^ and K_d_ of 1.9×10^−8^ M) make this engineered mutein a superb affinity agent for the purification of biotinylated biomolecules. Affinity matrices can be regenerated using gentle procedures, and regenerated matrices can be reused at least ten times without any observable reduction in binding capacity. With the combination of both the engineered mutein and wild-type streptavidin, biotinylated biomolecules can easily be affinity purified to high purity and immobilized to desirable platforms without any leakage concerns. Other potential biotechnological applications, such as development of an automated high-throughput protein purification system, are feasible.

## Introduction

Wild-type streptavidin is a tetrameric protein with four identical subunits. Each subunit has a biotin binding pocket and can bind biotin tightly with a dissociation constant (K_d_) around 10^−14^ M [1]. This binding is considered to be irreversible and has been applied in a wide range of biomedical and biotechnological applications [2], [3]. However, the tight biotin binding also makes streptavidin not suitable for affinity purification of biotinylated molecules. It would be ideal to develop engineered streptavidin muteins with reversible biotin binding capability. These engineered muteins can be applied to purify biotinylated molecules, develop automated high-throughput protein purification systems, reusable biosensor chips and bioreactors, study protein-protein interactions and design strippable probing agents (e.g. engineered muteins conjugated to horseradish peroxidase) for blot reprobing.

**Figure 1:**
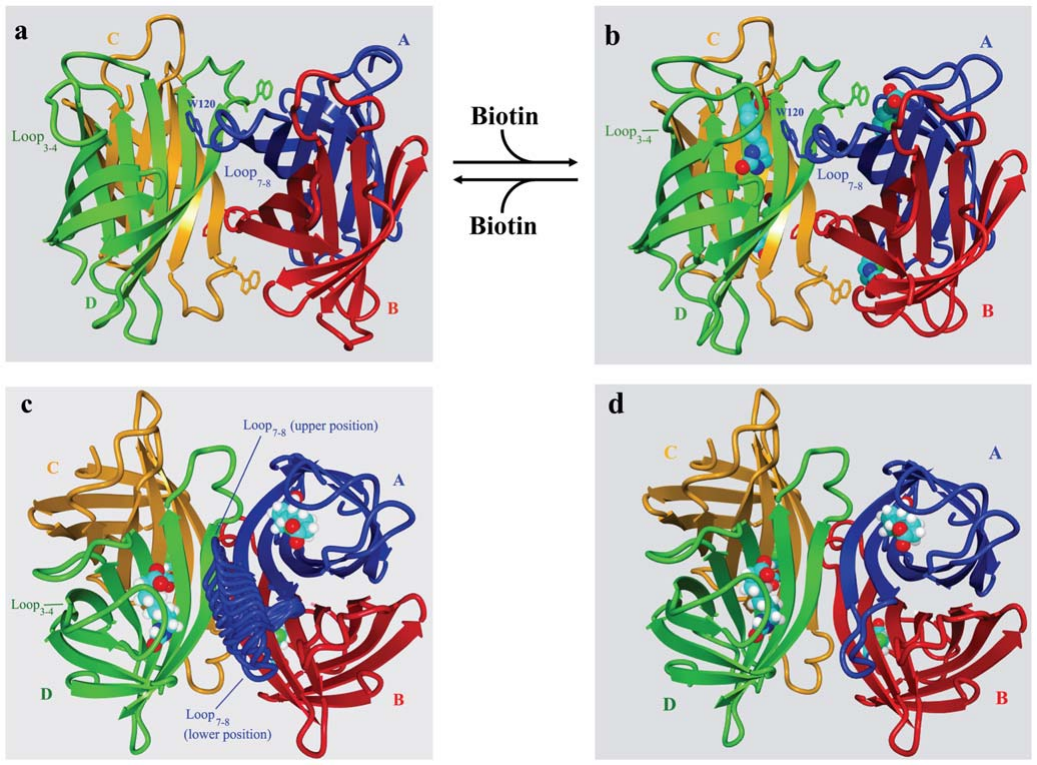
Biotin entry and exit paths in wild-type streptavidin (panels a and b) and models of the engineered mobile loop_7–8_ in streptavidin (panels c and d). Subunits A, B, C and D in wild-type streptavidin are colored in blue, red, yellow and green, respectively. Loop_3–4_ in subunit D is in the open conformation as shown in panel a to allow biotin binding. Trp-120 (blue) from subunit A (blue) is required to form a complete biotin binding pocket in subunit D (green). With biotin binding, loop_3–4_ is in the closed position as shown in panel b. To develop an engineered streptavidin (8-aa-loop-H127C) with reversible biotin binding, loop_7–8_ in wild-type streptavidin was replaced by an engineered mobile loop to function as a dynamic gateway for the exit of the bound biotin. The engineered mobile loop_7–8_ in subunit A is modeled in 10 different positions to illustrate its dynamic nature (panel c). The blue loop (loop_7–8_) in the upper positions will form a wall as part of the biotin binding pocket for the subunit D (green). At these stages, this gate is in the closed state. When loop_7–8_ is in the lower position (panels c and d), the gate is in the open state. With loop_3–4_ in subunit D in the closed conformation, biotin still can possibly escape from the biotin binding pocket at this stage (panel d).

To understand the strategies applied in engineering streptavidin with reversible biotin binding ability, it is vital to understand the structural features of streptavidin, its biotin binding pocket and the subunit interfaces. Each streptavidin subunit contains eight antiparallel strands that form a β-barrel structure [4], [5]. Two of these subunits (A and B as well as C and D in Fig. 1b) have extensive interfacial interactions to form a relatively stable dimer. Two dimers then assemble into a tetramer via a weaker interface. Although each subunit can bind a biotin molecule, each of the four complete biotin pockets in the tetramer relies on the donation of Trp-120 [6], [7], [8] located in loop_7–8_ from the neighboring subunit (e.g. subunit A needs Trp-120 from subunit D and vice versa, Figure 1b). Exceptionally tight biotin binding in streptavidin is mainly contributed by three sets of interactions [9]. The first set involves at least 6 residues (N23, S27, Y43, S88, T90 and D128) in the biotin binding pocket to form an extensive hydrogen bonding network with biotin. The second set involves strong hydrophobic interactions [7] between biotin and four tryptophan residues (79, 92, 108 and 120). Finally, residues in loop_3–4_ (S45, V47, G48, N49 and A50) play a critical role in binding and trapping biotin to the biotin binding pocket [10], [11], [12]. In the absence of biotin, loop_3–4_ has been shown to be flexible and is mainly in an open configuration. However, after biotin binding, loop_3–4_ becomes immobilized and is in a closed position (Figure 1, panels a and b) because of its interactions with biotin. Furthermore, biotin binding can also strengthen subunit interactions [13], in particular, via interactions between biotin in one subunit and Trp-120 in loop_7–8_ from the neighboring subunit [6]. In fact, the majority of the streptavidin-biotin complexes are in the tetrameric state in SDS-polyacrylamide gel even if the samples have been boiled before loading [14], [15].

Two approaches have been taken to develop streptavidin muteins with reversible biotin binding ability. The first approach is to replace amino acid residues that are critical in hydrogen bonding to biotin. Although these changes can indeed lower the biotin binding affinities [16], [17], many of the mutations also affect inter-subunit interactions in the streptavidin tetramer. Weakening inter-subunit interactions typically generates a heterogeneous population of streptavidin oligomers which leads to many practical problems. A second approach is to develop recombinant monomeric streptavidin [18] which has lower biotin binding affinity. This approach exploits the fact that an individual streptavidin subunit lacks a complete biotin binding pocket since Trp-120 from a neighboring subunit forms a key part of the binding site (Figure 1, panels a and b). Replacing Trp-120 with alanine (W120A) results in a tetrameric streptavidin mutein with a K_d_ of 3×10^−9^ M for biotin [17]. To create monomeric streptavidin, charge repulsion and steric hindrance were introduced at the subunit interface [18]. The resulting monomeric streptavidin has a biotin binding constant around 10^−7^ M. However, this mutein can be in the monomeric state only under certain conditions. First, the salt concentration has to be low to maximize electrostatic repulsion between streptavidin subunits. Second, the concentration of the monomers has to be low as exposure of the hydrophobic interface promotes non-specific aggregation.

In this study, a new approach was designed to develop an engineered tetrameric streptavidin with reversible biotin binding capability and other desirable features.

## Results

### Rationale for the design of novel streptavidin muteins

Our novel approach relies on the effects of two loops (loop_3–4_ with residues 45–52 and loop_7–8_ with residues 114–121) on biotin binding (Figure 1). Loop_3–4_ forms the lid and Trp-120 from loop_7–8_ forms part of the wall of the biotin binding pocket. With various hydrogen bonding and hydrophobic interactions in the biotin binding pocket and the closure of the lid, biotin can hardly escape from the binding pocket.

In this study, an attempt was made to create a dynamic “back door” formed by loop_7–8_ in streptavidin to allow biotin a second route to escape from the binding pocket when the “main door” primarily formed by loop_3–4_ is closed. This objective can potentially be achieved by several approaches. One is to develop a ΔW120 mutein. Since Trp-120 in loop_7–8_ is known to interact strongly with biotin [6], [7], [8], the deletion of Trp-120 may allow the modified loop_7–8_ to become more flexible. A second approach is to create a mobile 8-amino-acid-loop (8-aa-loop) mutein in which loop_7–8_ is engineered to have a completely different sequence but retains the same length as the original loop in wild-type streptavidin (Table 1). Asparagine and glycine were introduced at the center of the loop since they are known to introduce a turn in the loop structure [19]. The DSS (aspartate, serine and serine) and SDG (serine, aspartate and glycine) sequences were introduced to form the left and right arms of the loop, respectively (Table 1). These amino acids were selected because they have a high propensity for intrinsic disorder [20]. This design minimizes interactions between loop_7–8_ and biotin, and was intended to allow the engineered loop to act as an unlocked mobile door swinging between the open and closed states even in the presence of biotin (Figure 1, panels c and d). To improve the chance of obtaining a mutein with its engineered “back door” open wide enough for the exit of the bound biotin, a third approach was applied to create a series of muteins (2-aa-loop, 4-aa-loop and 6-aa-loop muteins, Table 1) with both the sequence of loop_7–8_ redesigned and the length of the loop shortened. Muteins with shorter loops were hypothesized to provide bigger openings around the biotin binding pocket which should facilitate the release of biotin. In a preliminary study, the 4-aa-loop mutein was immobilized on agarose matrix. Although this matrix could bind biotinylated proteins, streptavidin mutein was seen leaking from the column during washing and elution. Since the coupling condition allows on average one out of four subunits in the tetrameric streptavidin mutein to be coupled to the matrix to maximize the accessibility of the biotin binding sites, the leakage of streptavidin subunits suggests that changes in the loop_7–8_ structure might result in weakening of the inter-subunit interactions in the streptavidin mutein. To avoid this complication, the H127C mutation [21], [22] was introduced to two constructs (8-aa-loop mutein and ΔW120 mutein) to create 8-aa-loop-H127C mutein and ΔW120-H127C mutein, respectively. The H127C mutation has been reported [21], [22] to allow crosslink between subunits A and C (and also between subunits B and D) through the formation of a disulfide bond. These disulfide bonds can strengthen inter-subunit interactions. Since the matrix for the 4-aa-loop mutein had a subunit leakage problem, this mutein was not further characterized. The remaining four muteins (ΔW120-H127C, 2-aa-loop, 6-aa-loop and 8-aa-loop-H127C) were used for further analyses.

**Table 1:** Features of the loop_7–8_ muteins.

Streptavidin	Loop_7–8_	H127C
Wild-type	L T S G_113_ **T T E A N A W K** S_122_ T L V	−
ΔW120-H127C	**T T E A N A K**	+
8-aa-loop-H127C	**D S S N G S D G**	+
8-aa-loop	**D S S N G S D G**	−
6-aa-loop	**D S N G S G**	−
4-aa-loop	**D N G G**	−
2-aa-loop	**N G**	−

The amino acid sequences in the natural and engineered loop_7–8_ loops are shown in bold. The sequences flanking the loop_7–8_ are shown in black. + or − indicates the presence or absence of H127C in the muteins.

### Production and purification of streptavidin muteins

**Figure 2:**
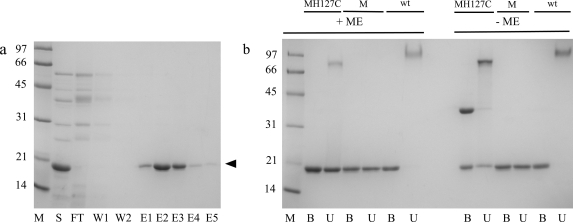
SDS-PAGE showing purification of 8-aa-loop-H127C mutein and its oligomeric state. In panel a, 8-aa-loop-H127C mutein was affinity purified from *B. subtilis* culture supernatant using biotin-agarose matrix. S represents the concentrated culture supernatant before purification. FT, W and E are the flow-through, wash and elution fractions, respectively. 5 µl from each fraction (800 µl per fraction, each fraction contained the same volume of sample) was reduced with β–mercaptoethanol and boiled before loading onto the gel. The arrowhead marks the position of the 8-aa-loop-H127C mutein. Panel b shows the tetrameric state of 8-aa-loop-H127C (MH127C). 8-aa-loop (M) and wild-type (wt) streptavidin were included as reference. B and U represent the boiled and unboiled samples respectively. ME represents β–mercaptoethanol (used at 0.7 M final concentration). M shows the position of the molecular weight markers (numbers on the left of both panels are expressed in kDa).

To avoid the formation of inclusion bodies and the need to refold the engineered streptavidin, all streptavidin muteins were produced in their soluble state from *B. subtilis* via secretion [23]. The production yield of the muteins in a semi-defined medium [24] was 40–60 mg/liter. Each mutein could be affinity purified in one step using the biotin-agarose matrix. The purification of the 8-aa-loop-H127C mutein showed typical results (Figure 2a). 8-aa-loop-H127C mutein was efficiently captured by the biotin-agarose matrix and could be eluted by buffer containing 4 mM biotin. The recovery was ∼90%. After dialysis to remove biotin from the pooled elution fractions, the purified mutein could rebind the biotin-agarose matrix and could be eluted again using biotin-containing buffer (data not shown). This result demonstrates the reversible biotin binding ability of the mutein.

### Determination of kinetic parameters

The kinetic parameters for biotin binding to various muteins were determined by surface plasmon resonance using the BIAcore biosensor with biotinylated IgG proteins as the ligand (Table 2). ΔW120-H127C had a binding affinity for biotin (K_d_ = 8.1×10^−9^ M) that was comparable to that of the W120A mutein (K_d_∼3×10^−9^ M) [17]. Although muteins with smaller loops (2- and 6-aa-loop muteins) were expected to have lower biotin binding affinities, their binding affinities (∼1–3×10^−9^ M) were actually comparable to that of the W120A mutein. Streptavidin muteins with nano-molar binding affinity tend to bind biotinylated molecules too tightly for affinity chromatography purification, leading to the poor recovery of target proteins. The mutein with the lowest biotin binding affinity (1.9×10^−8^ M) in this study is the 8-aa-loop-H127C mutein. It was further characterized and its ability to act as an affinity agent for purifying biotinylated proteins was explored.

**Table 2:** Kinetic parameters of biotin binding in streptavidin muteins.

Streptavidin mutein	ΔW120-H127C	2-aa-loop	6-aa-loop	8-aa-loop-H127C
k_a_ or k_on_ (M^−1^s^−1^)	4.96×10^4^	6.3×10^4^	2.43×10^4^	2.21×10^4^
k_d_ or k_off_ (s^−1^)	4.0×10^−4^	7.02×10^−5^	7.59×10^−5^	4.28×10^−4^
K_d_ (M)	8.1×10^−9^	1.12×10^−9^	3.13×10^−9^	1.9×10^−8^
t_½_ (min)*	28.9	165	152	27

Biotinylated IgG proteins were immobilized to the sensor chip, and purified muteins were injected to determine binding affinities.

* t_½_ is the half-life of biotin in the SAV-biotin complex. It is estimated based on the following equation: t_½_ (min) = 0.693/k_off_/60.

### Tetrameric state of 8-aa-loop-H127C mutein

Since the matrix with 4-aa-loop mutein immobilized showed leakage of streptavidin subunits during chromatography, this observation prompted the examination of the oligomeric state of the 8-aa-loop-H127C mutein. The migration pattern of the 8-aa-loop-H127C mutein resolved by SDS-PAGE was examined in the presence or absence of mercaptoethanol under boiled and non-boiled conditions (Figure 2b). 8-aa-loop mutein and wild-type streptavidin were included in the study as a comparison. Since the streptavidin inter-subunit interactions are weaker in the absence of biotin [6], these analyses were performed in the absence of biotin. Under all conditions, 8-aa-loop mutein migrated like a monomer. By contrast, over 98% of wild-type streptavidin remained as tetramers if the sample was not boiled. This suggests that changes in the amino acid sequence of loop_7–8_ weaken inter-subunit interactions. A significant difference in the oligomeric states of 8-aa-loop and 8-aa-loop-H127C muteins was observed in the absence of reducing agent. Whereas 8-aa-loop mutein remained as monomers whether the sample was boiled or not, ∼80% of 8-aa-loop-H127C mutein remained in the dimeric form even after boiling. This indicates the successful formation of disulfide bonds between subunits. With the unboiled sample, most of 8-aa-loop-H127C mutein adopted the tetrameric state. Since this mutein exists mainly as tetramer even in the presence of detergent in SDS-PAGE, it should be predominantly in the tetramer state under non-denaturing conditions. Thus, the H127C mutation did strengthen inter-subunit interactions in 8-aa-loop-H127C mutein.

### Purification of biotinylated protein G and biotinylated IgG using 8-aa-loop-H127C mutein-agarose matrix

**Figure 3:**
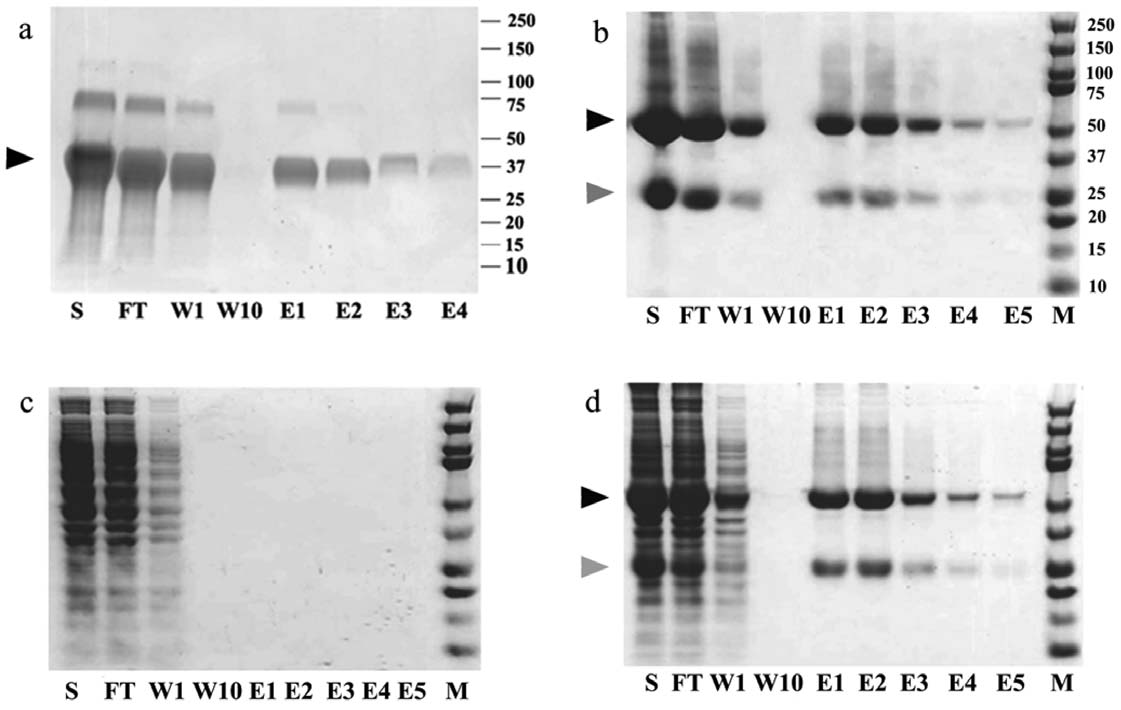
Purification of biotinylated protein G and IgG using 8-aa-loop-H127C mutein-agarose matrix. Excess amounts of biotinylated proteins were loaded to the column. Panel a: Biotinylated protein G with an apparent molecular mass of 37 kDa is marked by arrowhead. Some biotinylated protein G molecules are in oligomeric state with higher molecular masses. Panel b: The 54- and 25-kDa protein bands are the biotinylated heavy (marked by closed arrowhead) and light chains (marked by gray arrowhead) of IgG, respectively. Panel c: HeLa cell extract was applied to the 8-aa-loop-H127C mutein-agarose matrix. Panel d: Biotinylated IgG mixed with HeLa cell extract. S: Sample before purification, FT, W, and E: Flow-through, wash and elution fractions. M: Molecular weight markers.

To explore the feasibility of using 8-aa-loop-H127C mutein for the affinity purification of biotinylated molecules, chemically biotinylated protein G and IgG were captured on the 8-aa-loop-H127C mutein-agarose matrix (Figure 3, panels a and b). After removal of nonspecifically bound proteins, the bound biotinylated proteins could be eluted from the column using 4 mM biotin. Absence of a 20-kDa streptavidin band in the flow-through, wash and elution fractions suggested that streptavidin subunit leakage was not a problem. Estimation of the amount of IgG molecules captured on the column and quantification of biotinylated IgG in the elution fractions indicated the recovery to be approximately 95%. To demonstrate binding specificity, HeLa cell extracts were applied to the column. Non-specific binding was not observed (Figure 3c). Non-biotinylated protein G and IgG also could not bind to the matrix (data not shown). When biotinylated IgG was mixed with the HeLa cell extract, biotinylated IgG could be affinity purified to homogeneity in one step (Figure 3d).

To regenerate the column for repeated rounds of purification, the matrix was simply washed with 10 column volumes of binding buffer. Using biotinylated BSA as the target protein for purification, the 8-aa-loop-H127C mutein matrix saturated with excess amounts of biotinylated BSA could be regenerated in this manner to purify biotinylated BSA for 10 rounds with no observable loss in binding capacity (data not shown).

### Structural characterization of the 8-aa-loop-H127C mutein

**Figure 4:**
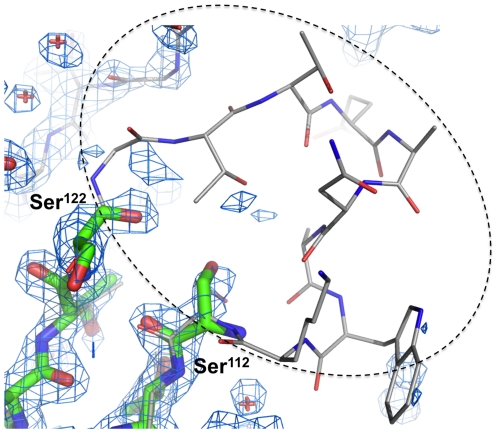
Electron density map (2|*F_o_*|-|*F_c_*| coefficients, contoured at 1.1 sigma) contoured around the region expected to be occupied by residues 114–121 of loop_7–8_. The model for residues 110–112 and 122–124 fit the electron density quite well, but there is insufficient electron density to model residues 113–121. The structure of wild-type streptavidin (PDB code 1SWE) is superimposed and drawn in a thinner, gray line representation. Coefficients and phases were calculated using Refmac and the figure was prepared using PyMOL.

To assess the conformation of the 8-aa-loop-H127C mutein, the protein was crystallized in the presence of biotin and its structure was solved using X-ray crystallography. Using data extending to 2.0 Å resolution, the structure clearly shows that the mutein crystallizes as a tetramer with a single subunit in the asymmetric unit (Figure S1). Biotin is bound in a manner indistinguishable from that of wild-type streptavidin, but there is very little electron density for loop_7–8_ (residues 114–121) and its neighboring residue 113 (Figure 4). The lack of electron density suggests that the modified sequence of loop_7–8_ confers a significant degree of dynamic disorder to the loop. The lack of interaction between loop_7–8_ and the biotin molecule bound to the adjacent subunit probably allows a higher level of mobility and dynamic motion in this loop in the mutein.

**Figure 5:**
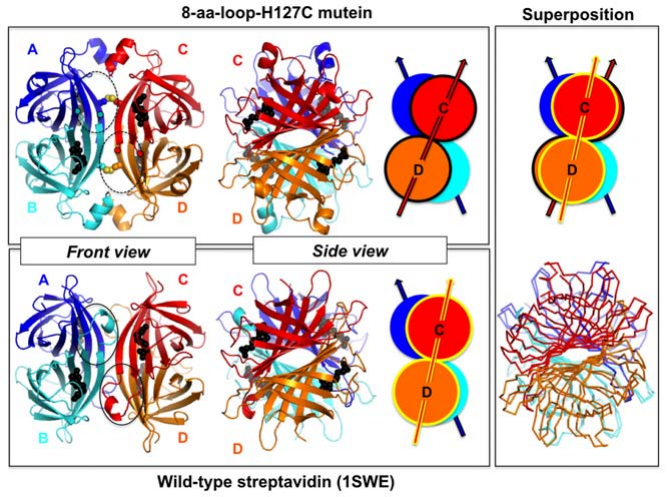
Comparison of quaternary structures of the 8aa-loop-H127C mutein and wild-type streptavidin (1SWE). Subunits A, B, C and D are colored blue, cyan, red and orange for both the mutein and wild-type structures. Biotin is drawn in space-filling representation and colored black. In all panels, only subunits A and B have been aligned. As a result, the cartoon diagrams and superimposed ribbon diagram at the far right show how the C/D dimer of the mutein is rotated by ∼10° relative to the C/D dimer of wild-type streptavidin when the A/B dimers of the mutein and wild-type streptavidin are superimposed. Loop_7–8_ (highlighted by the solid, black ovals in the front view) lies at the interface between dimer A/B and dimer C/D of wild-type streptavidin and is fixed in orientation in part by interactions between W120 and the biotin molecule bound to the opposing dimer. Loop_7–8_ is dynamically disordered in the mutein and hence is not shown in the cartoon model; the expected location of loop_7–8_ is denoted by the dashed, black ovals in the front view, and the location of the ends of the ordered parts of both loops (residues 112 and 122) are denoted by small circles. The side chains of Cys-127 from each subunit are drawn in space-filling representation, showing the formation of disulfide bonds between subunits A/C and B/D.

Since inter-dimer interactions are critical for stabilizing the tetrameric structure and will likely affect the dynamics of the streptavidin tetramer, changes introduced to the 8-aa-loop-H127C mutein can possibly affect the inter-dimer arrangement. In fact, a significant alteration in the arrangement of subunits in the 8-aa-loop-H127C mutein was observed when compared with wild-type streptavidin. The orientation of the A/B dimer relative to the C/D dimer appears to be very similar in nearly all of the previously reported structures of biotin-bound streptavidin [4], [5], partly because loop_7–8_ in one dimer interacts with a bound biotin molecule and nearby residues in the other dimer. Even when biotin is not present, Trp-120 and Leu-124 form mostly non-specific van der Waals contacts with Val-47 and Lys-121 from the opposing dimer, respectively. Thus, previously reported structures do not appear to have much difference in inter-dimer orientation or interactions. In this case, the loss of order in loop_7–8_ of 8-aa-loop-H127C mutein allows for a much larger rearrangement of the A/B dimer relative to the C/D dimer. When the A/B dimer of the mutein is superimposed onto the A/B dimer of wild-type streptavidin, the C/D dimer is rotated by 11° relative to the position of the C/D dimer in wild-type streptavidin (Figure 5). In contrast, the relative positions of the two dimers in other structures of biotin-bound streptavidin crystallized in different crystal forms differ by ∼1–3°. Significantly, an inter-dimer rotation of 5.4° was reported in the first paper comparing the structure of apo-streptavidin with biotin-bound streptavidin [4]. The even larger inter-dimer rotation seen in the 8-aa-loop-H127C mutein supports the correlation of a disrupted inter-dimer interface with less order in loop_7–8_ and lower biotin-binding affinity.

**Figure 6:**
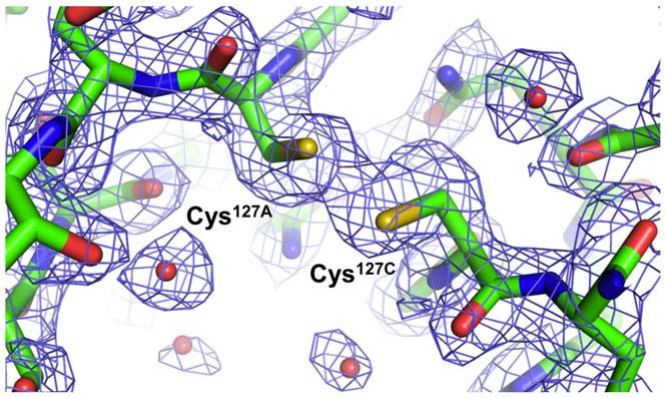
Electron density map (2|*F_o_*|-|*F_c_*| coefficients, contoured at 1.1 sigma) contoured around the model of the disulfide bond formed by Cys-127 residues from adjacent subunits (A and C or B and D in the tetramer shown in Figure 5). Coefficients and phases were calculated using Refmac and the figure was prepared using PyMOL.

An interesting feature also clearly seen in the crystal structure of the 8-aa-loop-H127C mutein is that a disulfide bond is formed between the Cys-127 residues of adjacent subunits at the A/B and C/D dimer interfaces (Figure 6). The Cys residue was introduced as a means of stabilizing the A/B and C/D dimers, based on the observed proximity of His-127 residues in adjacent subunits of the structure of wild-type streptavidin [4], [5], [21], [22]. The formation of this disulfide bond in the crystallized mutein is consistent with the presence of disulfide-bonded protein in non-reducing SDS-PAGE analysis as described above (Figure 2b).

## Discussion

An idealized streptavidin mutein for affinity chromatography applications should have the following desirable properties. First, it should be tetrameric and all four biotin binding sites are capable of binding biotin. In this state, the hydrophobic interface regions will not be exposed to the surface, thus minimizing non-specific hydrophobic interactions between the engineered streptavidin mutein and proteins in the crude sample to be analyzed. Second, intersubunit interactions should be strong. With one subunit of the tetramer immobilized, the other three subunits should stably associate with this covalently immobilized subunit so that no streptavidin subunits will be stripped off the column during the wash and elution steps. Third, the dissociation constant (K_d_) for biotin should be 10^−7^ to 10^−8^ M. Fourth, the off-rate (k_off_) for the bound biotin in the streptavidin-biotin complex is ideally around 10^−4^ sec^−1^ to allow the estimated half-life of the bound biotin to be around 10–30 minutes. With conditions 3 and 4 combined, the interaction would be both strong and specific enough to allow non-specifically bound molecules to be washed off the matrix without leakage of the specifically bound biotinylated molecules. At the same time, there should be efficient and quantitative elution of biotinylated molecules from the column. A fine balance between affinity towards biotinylated molecules and good recovery is essential for the ideal streptavidin affinity agent. Fifth, the engineered streptavidin muteins immobilized to the matrix should be stable enough to allow the matrix to be used for multiple rounds. Sixth, the engineered streptavidin should be produced with a reasonable production yield in a soluble and functional state without the requirement of refolding via inefficient and expensive denaturation and renaturation processes. The engineered 8-aa-loop-H127C mutein produced from *B. subtilis* via secretion meets all of the above requirements.

Replacement of residues in loop_7–8_ of wild-type streptavidin allows loop_7–8_ to become flexible and changes the relative orientation of subunits in the tetrameric structure. These structural changes can lead to the lowering of the biotin binding affinity in 8-aa-loop-H127C mutein. Introduction of the H127C mutation is for the objective to stabilize the mutein in the tetrameric state. To confirm that loop replacement is the major factor contributing to the observed lower biotin binding affinity in the 8-aa-loop-H127C mutein, the kinetic parameters of the 8-aa-loop mutein (without the H127C mutation) for binding biotinylated proteins were also determined (data not shown). Its dissociation constant (3.96×10^−8^ M) was found to be comparable to that (1.9×10^−8^ M) of the 8-aa-loop-H127C mutein. In contrary, the streptavidin mutein carrying solely the H127C mutation did not show a significant decrease in biotin binding affinity since it could not be eluted off from the biotin-agarose matrix using a biotin containing buffer.

The strength of biotin interactions with wild-type streptavidin and 8-aa-loop-H127C mutein was analyzed using the ligand energy inspector function in the Molegro molecular viewer program [25] based on X-ray crystallographic data. The binding free energy is estimated by the MolDock scores as shown in Table 3. The more negative values indicate stronger interactions. This analysis suggests that the lower biotin binding affinity in 8-aa-loop-H127C mutein is mainly contributed by both the absence of interactions (i.e. W120 and K121) and weaker interactions (D128, S45, N23 and L25) between biotin and residues in the biotin binding pocket. A previous study also suggests that D128, S45 and N23 play important roles in the biotin exit pathway [26]. Weakening the interactions between biotin and these residues is instrumental in enhancing biotin exit thereby increasing the biotin off rate (k_off_).

**Table 3:** Interaction of biotin with residues in the biotin binding pocket of subunit A.

Protein	8-aa-loop-H127C	Wild type SAV (2IZF)	Difference (wild type – mutein)
MolDock score	−125.6	−140.1	−14.4
**Trp 120 (from subunit D)**	0	−10.5	−10.5
**Lys 121 (from subunit D)**	0	−1.3	−1.3
**Asp128**	−4.4	−7.7	−3.3
**Ser 45**	−6.6	−8.0	−1.4
**Asn 23**	−4.1	−5.2	−1.1
**Leu 25**	−3.5	−4.3	−0.8

In addition to the interaction between biotin and specific residues in the biotin binding pocket of streptavidin, the strength of biotin binding appears to depend in part on the flexibility of loop_3–4_ and loop_7–8_. In both wild-type streptavidin [10] and the W120A mutein [7], both loops appear to be rigid in the presence of biotin. In contrast, the flexibility and mobility of loop_7–8_ in 8-aa-loop-H127C mutein creates a new exit path for the release of biotin from the biotin binding pocket even if loop_3–4_ is closed. This effect contributes to increases in the dissociation rate of the bound biotin.

During the course of this study, a new streptavidin mutein called traptavidin was reported [27]. Biotin dissociates from traptavidin at least 10 times slower than from wild-type streptavidin and binds with 10 times higher affinity. The crystal structure of traptavidin [28] indicates that loop_3–4_ is well-ordered and adopts a closed conformation in both the presence and absence of biotin. By contrast, loop_3–4_ is well-ordered only when biotin is bound to wild-type streptavidin. Because the conformation of the biotin-bound complex in traptavidin is virtually identical to that seen in wild-type streptavidin, the increase in biotin binding affinity is clearly not due to additional contacts between biotin and traptavidin, as supported by the analysis of binding interactions with Molegro, which assigns similar MolDock scores for biotin binding to traptavidin and wild-type streptavidin (−139.3 and −140.1 respectively). The increased rigidity of loop_3–4_ thus accounts for the higher biotin binding affinity in traptavidin. Conversely, the increased mobility of the engineered loop_7–8_ in 8-aa-loop-H127 mutein may in part account for the observed lower biotin binding affinity.

Most of the entire series of streptavidin muteins (ΔW120-H127C, 2- and 6-aa-loop muteins) have biotin binding affinities similar to that of the W120A mutein in the range of 10^−9^ M. Why the 2- and 6-aa-loop muteins do not have lower biotin binding affinities (K_d_>10^−8^ M) is unclear at present, but the dynamics of loop_7–8_ are likely important. Structural and molecular dynamics studies of these muteins may help to explain relative binding affinities.

Two matrices are commercially available to purify biotinylated molecules. One is the monomeric avidin matrix [29]. Unfortunately, this approach has three drawbacks. First, the production cost is high as three avidin subunits are sacrificed for each avidin subunit immobilized to the matrix. Second, the large hydrophobic surface exposed in the generation of monomeric avidin typically introduces problematic non-specific interactions with unwanted proteins in the crude extract. Third, it is difficult to completely denature the tetrameric avidin because of the strong subunit interactions. Incomplete denaturation of avidin results in the presence of some tetrameric avidin in the matrix which can bind biotinylated molecules irreversibly. The second commercially available matrix contains the engineered streptavidin mutein [17]. The main concern with this matrix is the occasional observation of leakage of streptavidin subunits during protein loading, washing and elution. With the above-mentioned limitations, the engineered 8-aa-loop-H127C mutein has substantial advantages over the currently available matrices for affinity purification of biotinylated molecules.

Many potential biotechnological applications of this mutein can be developed. In the post-genomic era with the discovery of many novel proteins which can be promising therapeutic targets, biomarkers for diseases, and agents with medical and biotechnological applications, the availability of a user-friendly high-throughput system to purify and immobilize these proteins for structural and functional studies is vital. However, protein purification requires reversibility in binding while immobilization requires ultra-tight interactions. Development of the 8-aa-loop-H127C mutein provides a solution to solve this dilemma. Biotinylated biomolecules can be affinity purified using the 8-aa-loop-H127C mutein matrix and immobilized to the wild-type streptavidin based protein chips. Furthermore, automated high-throughput purification platforms, reusable biosensor chips, protein arrays, bioreactors and magnetic beads can be developed using the 8-aa-loop-H127C mutein. With the reversible biotin binding capability, chemical conjugation or genetic fusion of 8-aa-loop-H127C muteins with other proteins such as alkaline phosphatase can allow blots to be reprobed without worrying masking other probing sites because of steric hindrance imposed by the bulky streptavidin conjugates.

Although the 8-aa-loop-H127C mutein in the present format has many desirable features for biotechnological application, two areas need further improvement. First, it would be ideal if its production yield can be increased further (>60 mg/liter). Since this mutein binds biotin reversibly, production of this mutein does not exert any toxic effect on its expression host by depleting biotin in both the cytoplasm and the culture medium. This is significantly different from the production of wild-type streptavidin which requires the production host to have the ability to produce sufficient quantities of biotin to sustain the cell growth [24]. Many other expression systems from prokaryotes to eukaryotes can now be explored. Second, a hybrid tag containing six histidine residues and a single cysteine residue [30] can be fused to either end of the 8-aa-loop-H127C mutein. When this mutein is produced at industrial scale, a single-step purification using biotin-agarose column is usually not sufficient to purify the mutein to homogeneity. The added his-tag can offer another round of affinity purification of this mutein via a different mechanism. The added cysteine residue in the tag can be applied for orientation specific coupling of the mutein to the thiol-based coupling column matrices, biosensor chips or protein arrays. This approach not only improves the accessibility of the biotin binding sites of the immobilized mutein in the affinity matrix [31] but also makes chemical coupling of this mutein to various matrices in a user friendly manner.

## Materials and Methods

### Construction of expression plasmids for production of streptavidin muteins in *B. subtilis*

Plasmid pSSAV [23] carrying a *B. subtilis* levansucrase signal peptide for secretion and a P43 promoter for transcription was used as the expression vector. This vector carries a synthetic gene for wild-type full-length streptavidin. To construct the expression vectors for the loop muteins, the gene encoding the wild-type streptavidin counterpart in pSSAV was replaced by the *Pst*I/*Bcl*I synthetic fragments encoding the loop muteins. *E. coli* pbluescript plasmids containing the synthetic gene fragments were ordered from Epoch Life Science, Inc. Texas, U.S.A. Each *E. coli* plasmid was digested with *Pst*I/*Bcl*I to release the synthetic gene fragment which would then be ligated to the *Pst*I/*Bcl*I cut pSSAV to generate the expression vectors.

### Production and affinity purification of streptavidin muteins

*B. subtilis* WB800 [32] cells carrying the expression vectors were cultivated in a semi-defined medium [24] at 30°C for 9–12 hours. Culture supernatant was collected by centrifugation and streptavidin muteins were affinity purified using biotin-agarose as described previously [18].

### X-ray crystallographic studies

Crystals of 8-aa-loop-H127C mutein were obtained using the hanging-drop vapor diffusion technique by combining 2 µL protein (4 mg/mL, 10 mM Tris-Cl pH 7.5, 150 mM NaCl) with 2 µL reservoir solution (50% Tacsimate, pH 6.0 (Hampton Research), 10% (w/v) glycerol). Crystals grew to an approximate size of 0.2×0.2×0.1 mm after ∼2 weeks. They were suspended in a microfabricated loop (Mitegen) and flash-cooled under a nitrogen gas stream (∼100 K, Oxford Cryosystems). Data were measured using X-rays from a rotating anode generator (Rigaku RU-H3R), Multilayer optics (Osmic Blue) and Mar345 imaging plate detector (MarResearch). Diffraction data (200 images×0.5°; 10 minutes/image) were indexed and scaled using DENZO and Scalepack [33]. The space group was assigned by autoindexing, inspecting systematic absences and scaling. It was confirmed to be P4_2_2_1_2 by molecular replacement calculations and refinement. PHASER [34] was used for molecular replacement calculations, yielding a single very strong solution using a single subunit of wild-type streptavidin (1SWE) as the search model. Refinement calculations were performed using REFMAC (v. 5.5.0109) [35], and model building was performed using COOT (v. 0.6.1) [36]. Geometric parameters were monitored using Procheck [37] and Molprobity [38]. Crystallographic statistics are reported in Table 4.

**Table 4:** Crystallographic statistics.

*Data collection*	
Space Group	
Mol/A.U.	1
Unit cell a, b, c (Å)	57.1, 57.1, 70.2
Resolution (Å)^a^	20.0−1.95 (2.02−1.95)
R_sym_ b	0.063 (0.496)
I/σI	28.4 (2.75)
Completeness (%)	96.1 (88.0)
Redundancy	6.7 (3.6)
*Refinement*	
Resolution (Å)	19.39−2.00
Unique reflections	7585
R_work_ c/R_free_ d	0.189/0.232
No. of atoms	1003
Protein atoms	915
Biotin	16
Water	72
r.m.s.d. from ideal^e^ geometry	
Bond lengths (Å)	0.009
Bond angles (°)	1.26
Ramachandran angles^f^	
%-favored	97.5%
%-outliers	0%

a Values from the outermost resolution shell are given in parentheses.

b R_sym_ = Σ_i_|I_i_−<I>|/Σ_i_ I_i_ where I_i_ is the *i*th integrated intensity of a given reflection and <I> is the weighted mean of all measurements of I.

c R_work_ = Σ||F_o_|−|F_c_||/Σ|F_o_| for 95% of reflection data used in refinement.

d R_free_ = Σ||F_o_|−|F_c_||/Σ|F_o_| for 5% of reflection data excluded from refinement.

e Root-mean-square deviations from ideal geometry calculated by Procheck [37].

f Ramachandran plot analysis carried out using Molprobity [38].

### Analysis of interactions between biotin and streptavidin using Molegro viewer

The pdb files of the biotin complexes associated with 8-aa-loop-H127C mutein, wild-type streptavidin (2IZF) and traptavidin (2Y3F) [28] were used as the input files. Each of these files was imported to Molegro Molecular Viewer (Version 2.2.0) [25]. Interaction energy analysis was performed using the ligand energy inspector module in the program. Before analysis, both the ligand and protein hydrogen bonding positions were optimized and the ligand was energy minimized using the action panel in the ligand energy inspector module. The protein-ligand interaction energy (E_inter_, sum of the steric interaction energy, hydrogen bonding energy, short- and long-range electrostatic interaction energies) was expressed in the form of the MolDock score [25] in arbitrary units. A more negative value reflects a stronger interaction.

### Other methods

Determination of kinetic parameters using BIAcore biosensor, preparation of streptavidin-agarose matrix and purification of biotinylated proteins were performed as previously described [18], [39]. The affinity column had a bed volume of 0.1 ml 8-aa-loop-H127C mutein coupled matrix. 200 µl sample containing 0.2 mg of biotinylated protein G or 0.44 mg biotinylated IgG from ThermoFisher was applied to the column. To purify biotinylated IgG from a mixture, HeLa cell extract containing 0.6 mg protein was mixed with 0.44 mg biotinylatyed IgG. This mixture was then applied to the affinity column for purification. The graphic drawings of the structure of the streptavidin-biotin complex shown in Fig. 1 were generated by using the Yasara program [40], (YASARA Biosciences GmbH, Austria). To model the possible positions of loop_7–8_, the pdb file (1SWE) of the streptavidin-biotin complex was used as the input file. The amino acid residues (G113 and S122) at the base of loop_7–8_ were fixed. The possible conformations of loop were searched against the protein data bank using the loop modeling module in Yasara. Two modeled structures with loops in the upper and lower positions were selected as the input pdb files. These two input structures define the boundary of the loop movement. A pdb file containing 10 modelled structures generated from the Morph server [41] website was analyzed by Yasara to generate the animated structures shown in Fig. 1c.

## Supporting Information

Figure S1 A complete tetramer of the 8aa-loop-H127C mutein is drawn, which each subunit drawn in a different color. The unit cell is drawn as a black box. The two-fold axes running through the origin are drawn according to standard crystallographic conventions. Three orthogonal views are drawn: each view is parallel to one of the three crystallographic axes. For each view, the two remaining axes orthogonal to axis being viewed down are labeled.
(DOCX)
