# Supplementary figures and images for: Development of a Tetrameric Streptavidin Mutein with Reversible Biotin Binding Capability: Engineering a Mobile Loop as an Exit Door for Biotin
Source: PLoS One. 2012 Apr 19;7(4):e35203. doi: 10.1371/journal.pone.0035203 (PMC3334968; doi:10.1371/journal.pone.0035203)

Figure S1


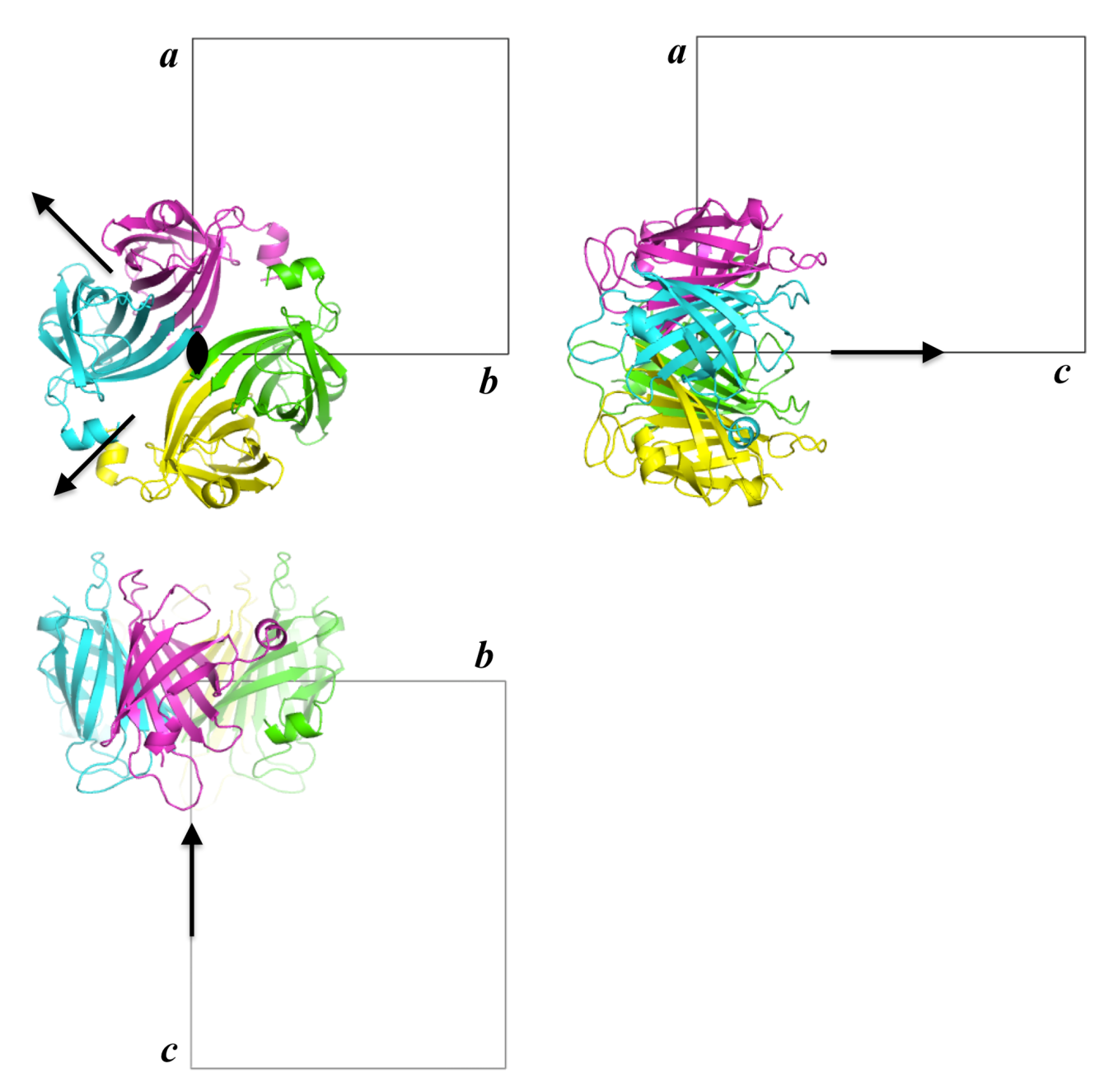

Supplement: Figure S1 — A complete tetramer of the 8aa-loop-H127C mutein is drawn, which each subunit drawn in a different color. The unit cell is drawn as a black box. The two-fold axes running through the origin are drawn according to standard crystallographic conventions. Three orthogonal views are drawn: each view is parallel to one of the three crystallographic axes. For each view, the two remaining axes orthogonal to axis being viewed down are labeled. (DOCX) [file pone.0035203.s001.docx]
